# It Is Not Much

**Published:** 1893-02

**Authors:** 


### IT IS NOT MUCH.

It is not much that I can do
In this great world of ours.
It is not much! but well I know,
The tiny drops that fall in showers,
From thready streams to rivers grow,
And mighty oceans form below.

And may not I in ways obscure
Some little seeds of kindness sow,
That they may reap the harvest sure,
Whose greater needs from hardships flow,
And thus life's thorny pathway make
A little smoother for their sake?

—LA CROIX.

